# Rates of maximal blood lactate accumulation in the extreme intensity exercise domain: implications for training prescription

**DOI:** 10.1007/s00421-026-06290-x

**Published:** 2026-06-29

**Authors:** Ozgur Ozkaya, Hakan As, Arda Peker, Mark Burnley, Andrew M. Jones

**Affiliations:** 1 Department of Coaching Education, Faculty of Sports Sciences, Ege University, 35050 Bornova, Izmir, Turkey; 2 AixTech Performance Lab, Ege University Technopark, Bornova, Izmir, Turkey; 3 School of Sport, Exercise and Health Sciences, Loughborough University, Leicestershire, UK; 4 Faculty of Health and Life Sciences, University of Exeter Medical School, Exeter, UK

**Keywords:** All-out sprint, Breakpoint in *v*La, Cycle ergometry, Maximal lactate formation rate, V̇O_2_ kinetics, *v*La_max_

## Abstract

**Purpose:**

This study investigated the rate of blood lactate accumulation (*v*La) across six maximal sprint efforts and identified a breakpoint (*v*La_BP_) in the *v*La–power relationship.

**Methods:**

Twelve physically active males completed 5, 10, 15, 20, 25, and 30-s all-out sprints in randomised order on separate days, with resistances set at 10 to 12.5% of body mass. The *v*La was calculated as the change in blood lactate between the resting and peak value (∆La) divided by the test duration. A piecewise linear regression was used to identify the *v*La_BP_ and its corresponding power output (P-*v*La_BP_) in the *v*La–power relationship. Pulmonary V̇O_2_ responses were modelled using V̇O_2_ data obtained from exercise and the subsequent 15-min recovery period.

**Results:**

A distinct P-*v*La_BP_ was identified at 795 ± 135 W and 11.2 ± 1.5 W·kg^−1^, corresponding to a *v*La of 0.50 ± 0.10 mM·s^–1^, ∆La of 6.50 ± 1.27 mM, and sprint duration of 13.3 ± 1.9 s. The *v*La did not increase significantly during the 30, 25, 20, and 15-s all-out sprint tests (0.41 ± 0.04 to 0.48 ± 0.10 mM·s⁻^1^; *p* > 0.05) but increased markedly in the 10 and 5-s tests (0.58 ± 0.14 mM·s⁻^1^, *p* < 0.05; 0.7 ± 0.20 mM·s⁻^1^, *p* < 0.01). V̇O_2_ on-kinetics below the P-*v*La_BP_ (30 and 15 s) was best described by linear function (R^2^ ≥ 0.96; SEE ≤ 272 mL·min^–1^; *p* < 0.001), while the V̇O_2_ data could not be modelled for sprints performed above the P-*v*La_BP_ (10 and 5 s).

**Conclusion:**

The present study demonstrated that the P-*v*La_BP_ separates ranges of power outputs with discrete V̇O_2_ kinetics which are characteristic of different subzones within the extreme intensity exercise domain.

## Introduction

Within the exercise sciences, exercise intensity domains are typically classified as moderate, heavy, severe, and extreme (Hill et al. 2002; Poole et al. 2005; Burnley and Jones 2007; Poole and Jones 2012; Bishop et al. 2025). The moderate, heavy and severe intensity exercise domains are characterised by distinct dynamic behaviours of V̇O_2_ kinetics and blood lactate concentration (La), and these behaviours are consistent within each domain even at different absolute power outputs (Whipp and Mahler 1980; Gaesser and Poole 1996; Poole et al. 2005; Burnley and Jones 2007). However, V̇O_2_ and blood La kinetics have been less well characterised for the extreme intensity exercise domain. This is surprising given that, for a healthy and physically active individual, the extreme intensity exercise domain incorporates a potential range of power outputs almost twice as large as the sum of the moderate, heavy, and severe intensity domains (Ozkaya et al. 2023).

Notably, a recent study reported that V̇O_2_ kinetics change significantly as the power output increases within the extreme intensity exercise domain, and recognised three subzones, termed extreme-1, extreme-2 and extreme-3 (Ozkaya et al. 2023). Based on this framework, the V̇O_2_ kinetics in the extreme-1 intensity exercise zone is exponential, but it is not possible to attain maximal O_2_ uptake (V̇O_2max_) at task failure. In the extreme-2 zone, V̇O_2_ kinetics are best described by a linear function, while V̇O_2_ cannot be modelled due to the very brief exercise duration in the extreme-3 zone. Given the breadth of the extreme intensity domain, it has been suggested that these sub-categories may help in understanding physiological responses to exercise and in guiding training. Nevertheless, although a subzone approach within the extreme intensity exercise domain has been proposed, supported by discrete V̇O_2_ on- and off-response kinetics, methods for determining the transition intensities (i.e., as the power output or running speed) that separate the subzones within the domain have not yet been established.

Recently, there has been increasing interest in the rate of blood lactate accumulation (*v*La) during short-term maximal exercise and especially its potential role in the evaluation of athletes’ performance (Quittmann et al. 2020, 2021; Yang et al. 2023; Dunst et al. 2023; Meixner et al. 2024a, b; Langley et al. 2024; Thron et al. 2024; Quittmann 2026). It has been reported that end-exercise peak blood La obtained from 5–10-s maximal efforts is significantly lower than that measured following 30–60-s sprint durations (Heck et al. 2003; Dunst et al. 2023; Langley et al. 2024). However, those 5–10-s short sprint bursts lead to a higher maximal lactate accumulation rate (*v*La_max_) (Mavroudi et al. 2023; Langley et al. 2024; Porter and Langley 2025; Quittmann 2026). If there is an inverse relationship between longer sprint durations that elicit a higher end-exercise peak blood La response and shorter sprint durations that elicit a higher *v*La response, this might be detectable as a breakpoint in the *v*La–power relationship (*v*La_BP_). This breakpoint would occur at a specific power output (P-*v*La_BP_) and have a corresponding specific sprint duration (t-*v*La_BP_). It is possible that this P-*v*La_BP_ may represent a transition between exercise in which there are adequate V̇O_2_ data that may be described by a linear function (i.e., extreme-2) and exercise in which V̇O_2_ cannot be modelled due to the very brief exercise duration dominated by breath-holding consistent with a Valsalva manoeuvre (i.e., extreme-3).

The aim of this study was, therefore, to investigate blood La dynamics during recovery from maximal sprint exercise of different durations and to calculate the corresponding *v*La. We hypothesised that plotting the *v*La values against the corresponding mean power outputs might reveal a P-*v*La_BP_, which separates the extreme-3 from extreme-2 subzone based on both blood La dynamics and V̇O_2_ kinetics.

## Materials and methods

### Participants

This study received ethical approval from the Ege University Ethics Committee (Protocol no: 22-2T/16, 2 October 2022) and conformed to the principles outlined in the Declaration of Helsinki (1983). Prior to participation, all procedures, along with potential risks and benefits, were clearly explained to the volunteers, and written informed consent was obtained in accordance with the approved documentation. The study recruitment was open to both sexes, but only male participants volunteered to take part. A total of twelve healthy, physically active males took part in the study (mean ± SD: age: 26.6 ± 6.6 years; height: 1.76 ± 0.07 m; body mass: 71.3 ± 12.9 kg). None of the participants reported any cardiovascular, pulmonary, metabolic or neuromuscular disorders. Participants were instructed to maintain consistent dietary habits throughout the study period, refrain from strenuous exercise in the 24 h preceding each testing session and avoid caffeine or alcohol intake during this same time frame.

### Experimental overview

This study utilised a counter-balanced, randomised, within-subject crossover (repeated measures) design to examine different sprint durations, integrating blood lactate measurements, *v*La–power relationships, and V̇O_2_ on- and off-kinetics. The study protocol comprised eight laboratory visits per participant, each separated by 24–48 h, and was completed over a period of 2–3 weeks. A familiarisation session was undertaken before the sprint trials to minimise single practice effects. Following a ramp incremental exercise test, each participant performed six all-out sprint tests (i.e., 5, 10, 15, 20, 25, and 30 s) on separate days. The calculated *v*La values were plotted against the corresponding power outputs to define a breakpoint (i.e., *v*La_BP_) in the relationship. The *v*La_max_ was also determined for each participant. Time-dependent V̇O_2_ responses were modelled to describe V̇O_2_ kinetics. All exercise tests were conducted in a well-ventilated laboratory, under standardised conditions (20–22 °C temperature, ~20.8% O_2_, ~0.05% CO_2_, and 50–60% relative humidity). The time of the day allocated for testing was also standardised (1.5 ± 0.4 h) to control for diurnal variance.

### Procedures

#### Ramp incremental tests

Ramp incremental tests were performed on an electromagnetically braked cycle ergometer (Monark LC7, Vansbro, Sweden) with a sampling interval of 1 Hz. The manufacturer certifies that measurement error is below 2% when the ergometer is regularly calibrated. Based on previous studies indicating that an optimal knee angle at the pedal’s bottom dead centre position is approximately 150° (Bohlmann 1981; de Vey Mestdagh 1998), the seat height was adjusted to provide a knee angle of 150° for each participant. Ramp incremental tests commenced with 4 min of cycling without resistance before the power output was increased by 30 W·min^−1^. Participants were asked to maintain their preferred cadence for as long as possible (i.e., approximately 70–90 rpm). The tests were terminated when participants’ cadence fell more than 10 rpm below their preferred cadence for longer than 10 s, despite verbal encouragement. Pulmonary gas exchange and ventilation data were measured breath-by-breath throughout the ramp tests using a metabolic cart (Cosmed Quark CPET, Rome, Italy). Data were initially filtered to remove occasional errant breaths resulting from e.g., swallows, coughs, or sighs. V̇O_2_ data points lying more than four standard deviations from the local mean (five breaths) were deleted and were smoothed using a 5-breath moving average. Data were then exported in 5-s binned averages. The highest 30-s moving average of V̇O_2_ (i.e., V̇O_2max_) and the corresponding power output (i.e., P-V̇O_2max_) were determined for each participant.

#### Familiarisation sessions

Familiarisation sessions for the all-out sprint tests were performed on a mechanically braked cycle ergometer (Monark Peak Bike 894E, Varberg, Sweden). During these sessions, all participants accelerated the flywheel to its maximum speed, and 7–8 s sprints were performed without additional frictional resistance. The performance criterion in this first stage of familiarisation was reaching peak flywheel speed within 5–6 s depending on flywheel inertia (Ozkaya 2013). Participants who met the criterion in two consecutive trials were given 5 min of rest before proceeding. In the second stage of familiarisation, following the pre-acceleration phase, a load corresponding to 10% of body mass was applied to the system, once the cadence reached 120 rpm. Subsequent all-out trials were considered successful when the time to peak power ≤ 2 s was achieved in two consecutive trials (Ozkaya 2013). Participants fulfilling these criteria were deemed to be familiarised and were admitted to the all-out sprint sessions.

#### All-out sprint sessions

Since we know that a traditional Wingate-type 30-s sprint is characterised by a linear behaviour in V̇O_2_ kinetics (Beneke et al. 2002; Ozkaya et al. 2014) and is typically performed within the extreme-2 intensity exercise zone (Ozkaya et al. 2023), the longest all-out sprint test duration was set to 30-s Wingate-type task. In this phase of the study, each participant completed six all-out sprint tests (5, 10, 15, 20, 25, and 30 s) on separate days in a randomised cross-over design. To obtain valid *v*La estimations, resistance was systematically increased by 0.5% over the typical Wingate load (i.e., 10% of body mass) for each test, and all tests were performed all-out without pacing. Body mass was measured before each testing session using a calibrated digital scale (Seca 217, Hamburg, Germany) with participants wearing minimal clothing and no shoes. Accordingly, the resistance applied for 30, 25, 20, 15, 10, and 5-s sprints corresponded to 10%, 10.5%, 11%, 11.5%, 12%, and 12.5% of body mass, respectively. Participants completed a 5-min warm-up at ~100 W at their preferred cadence (70–90 rpm), followed by a 5-min rest. Each test then began with 4 min of no-resistance cycling at ~50 rpm. During the final 30 s of the baseline period, participants stopped pedalling and awaited the start command. All tests were performed in a seated position, starting from a static position with the dominant leg at the top of the first pedal stroke. A verbal countdown (“3–2–1–go”) signalled the start of each sprint, at which point participants were encouraged to produce maximal effort from the first pedal stroke. Once cadence exceeded 120 rpm, the ergometer automatically applied the pre-set resistance (i.e., 10 to 12.5% of body mass), initiating data acquisition. Participants were verbally encouraged to maintain maximal effort throughout the sprint. Power output (i.e., W and W·kg^−1^) and cadence (i.e., rpm) were recorded continuously. The highest instantaneous power production during the sprint was considered the P_max_, while the mean power output measured during each sprint was recorded as the P_avg_. The highest 30-s moving average of V̇O_2_ measured during the sprints and the following 15-min recovery periods were also analysed from 5-s binned averages.

#### Estimation of the *v*La and *v*La_max_

Blood La values were analysed, using 20-μl blood samples, which were collected from the fingertip before, at the end, and at the 1st, 3rd, 5th, 7th and 10th min of the recovery periods, by the enzymatic amperometric method (Biosen C-line, EKF Diagnostics, GmbH, Barleben, Germany). A resting blood La sample was obtained upon arrival at the laboratory, before the warm-up, after ≥ 5 min of seated rest. The change in blood La (i.e., ∆La in mM) between the resting (La_rest_) and post-exercise peak La values (La_peak_) obtained from all six sprint tests were calculated. The *v*La (in mM·s^–1^) was considered the ∆La divided by the sprint duration (t_test_) (Eq. 1). *v*La_max_ was defined as the highest *v*La value obtained across the six sprint tests.

1 $$v{\mathrm{La}} = \frac{{{\mathrm{La}}_{{{\mathrm{peak}}}} - {\text{ La}}_{{{\mathrm{rest}}}} }}{{{\mathrm{t}}_{{{\mathrm{test}}}} }}$$

where *v*La is the lactate accumulation rate; La_peak_ is the peak blood La response to each sprint obtained from post-exercise measurements; La_rest_ is the resting blood La measurement; and t_test_ is the sprint duration. Note that the *v*La_max_ was considered the highest *v*La value obtained across the six sprints.

#### Estimation of breakpoints in the relationship between *v*La and power output

Calculated *v*La values (on the y-axis) were plotted against the P_avg_ during the sprints (on the x-axis) to derive a *v*La_BP_ for each participant. A two-segment linear regression model (SigmaPlot; Systat Software Inc., San Jose, CA) was used to mathematically determine the *v*La_BP_. Regression analysis essentially fits two separate linear regression lines to different domains of the data defining the gradient of the first and second segment, the change in gradient from the first to second segments, and the breakpoint position (Muggeo 2003). The power output at the breakpoint position (i.e., P-*v*La_BP_) and the exercise duration corresponding to the P-*v*La_BP_ (i.e., t-*v*La_BP_) were then recorded for each participant individually. Breakpoint positions which were determined statistically were then visually verified by five sports physiologists, who were asked to independently determine breakpoint positions in the *v*La–power output relationships (Ozkaya et al. 2025).

#### Analysis of pulmonary V̇O_2_ kinetics

The breath-by-breath V̇O_2_ data were initially modelled using a mono-exponential function. However, this approach failed to provide an adequate fit, likely due to the rapid termination of the exercise. In contrast, the V̇O_2_ kinetics was more appropriately described by a linear model, which yielded a superior goodness of fit (Ozkaya et al. 2023) (Eq. 2):

2 $${\dot{\mathrm{V}}\mathrm{O}}_{2\left( t \right)} = {\dot{\mathrm{V}}\mathrm{O}}_{{2\left( {\mathrm{base}} \right)}} + {\mathrm{at}}$$

where V̇O_2(t)_ is the V̇O_2_ responses achievable at any time during exercise; V̇O_2(base)_ is the baseline V̇O_2_ during the last 1 min of no-resistance cycling; and a is the slope of the line.

Furthermore, post-exercise V̇O_2_ kinetics was described by exponential decay models based on data obtained from 15-min recovery period. Thus, the behaviour of the V̇O_2_ kinetics over time was fitted by single and double exponential decay models (Eqs. 3 and 4):

3 $${\dot{\mathrm{V}}\mathrm{O}}_{{2\left( {\mathrm{t}} \right)}} = {\dot{\mathrm{V}}\mathrm{O}}_{{2\left( {{\mathrm{base}}} \right)}} + {\mathrm{A}}_{{{\mathrm{fc}}}} \cdot {\mathrm{e}}^{{ - \left( {{\mathrm{t}} - {\mathrm{TD}}_{{{\mathrm{fc}}}} } \right)/_{{{\mathrm{τfc}}}} }}$$

where V̇O_2(t)_ is the V̇O_2_ response achieved at any time during post-exercise; V̇O_2(base)_ is the baseline V̇O_2_ during the last 1 min of no-resistance cycling; and A_fc_ is the amplitude of the fast component, τ_fc_ is the time constant of the fast component, and TD_fc_ is the time delay of the post-exercise V̇O_2_ response.

4 $$\begin{aligned} {\dot{\mathrm{V}}\mathrm{O}}_{{2\left( {\mathrm{t}} \right)}} & = {\dot{\mathrm{V}}\mathrm{O}}_{{2\left( {{\mathrm{base}}} \right)}} \\ & \quad + {\mathrm{A}}_{{{\mathrm{fc}}}} \cdot {\mathrm{e}}^{{ - \left( {{\mathrm{t}} - {\mathrm{TD}}_{{{\mathrm{fc}}}} } \right)/_{{{\mathrm{τfc}}}} }} + {\mathrm{A}}_{{{\mathrm{sc}}}} \cdot {\mathrm{e}}^{{ - \left( {{\mathrm{t}} - {\mathrm{TD}}_{{{\mathrm{sc}}}} } \right)/_{{{\mathrm{τsc}}}} }} \\ \end{aligned}$$

where V̇O_2(t)_ is the V̇O_2_ response achieved at any time during post-exercise; V̇O_2(base)_ is the baseline V̇O_2_ during the last 1 min of no-resistance cycling; A_fc_ is the amplitude of the fast component, τ_fc_ is the time constant of the fast component, and TD_fc_ is the time delay of the fast component; and A_sc_ is the amplitude of the slow component, τ_sc_ is the time constant of the slow component, and TD_sc_ is the time delay of the slow component.

Linear, single and double exponential decay models were applied to describe the V̇O_2_ kinetics using Sigma-Plot 14.0 software (Systat Software Inc., San Jose, CA, USA). Regression analyses were used to test the fitting behaviour of the V̇O_2_ data based on the standard error of the estimate (SEE) and the coefficient of determination (R^2^). On- and off- V̇O_2_ kinetics, which could not be modelled due to very short exercise durations in response to very high-power outputs, were assessed visually by five sports physiologists, who were asked to independently determine the V̇O_2_ shape over time (Ozkaya et al. 2023).

### Statistical analyses

All statistical analyses were performed using SPSS Statistics 26 (IBM Corp, Armonk, NY, USA). Normality was tested with the Shapiro-Wilk test, and homogeneity of variance was assessed by Levene’s test. Sphericity was evaluated using Mauchly’s test: when assumptions were violated, degrees of freedom were corrected using Greenhouse-Geisser (ε < 0.75) or Huynh-Feldt (ε > 0.75) as appropriate. Differences in power output, V̇O_2_, La, and time related variables across sprint tests were then analysed using a repeated-measures ANOVA. Pairwise comparisons were conducted with Bonferroni adjustment for multiple comparisons. Effect size (ES) values for repeated measures ANOVA were computed using partial eta squared (${\upeta}_{\mathrm{p}}^{2}$) and categorised as follows: (i) small ES when 0.04 < ${\upeta}_{\mathrm{p}}^{2}$ ≤ 0.25; (ii) moderate ES when 0.25 < ${\upeta}_{\mathrm{p}}^{2}$ ≤ 0.64; and (iii) large ES when ${\upeta}_{\mathrm{p}}^{2}$ > 0.64 (Ferguson 2009). A Pearson product-moment correlation was computed to evaluate the relationship between ∆La and *v*La derived from the sprint tests. Correlation magnitudes were interpreted using the qualitative thresholds proposed by Hopkins et al. (2009): trivial: (r < 0.10), small (0.10 ≤ r < 0.30), moderate (0.30 ≤ r < 0.50), large (0.50 ≤ r < 0.70), very large (0.70 ≤ r < 0.90), and nearly perfect (r ≥ 0.90). Significance levels were set at p ≤ 0.05. Data are presented as mean ± SD.

## Results

**Fig. 1:**
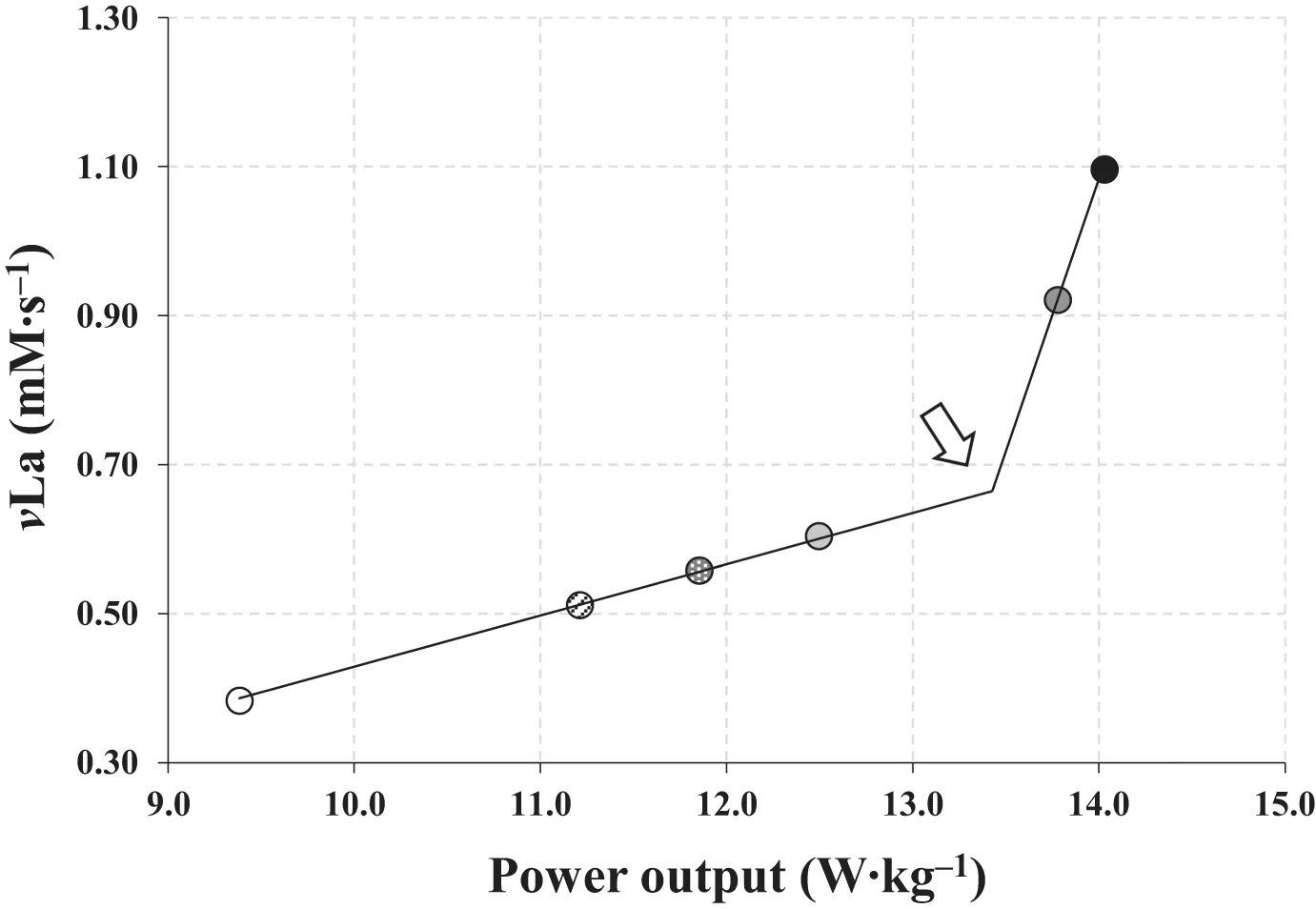
Assessment of the breakpoint in the relationship between the rate of blood lactate accumulation (*v*La) and power output derived from six all-out sprints (from 30 to 5 s) in a representative participant. The *v*La_BP_ was determined as the intersection of the two regression lines (black arrow). Data points are shown with a fill gradient from light to dark colour in proportion to the decreasing sprint durations

The mean time to limit of tolerance at the end of the ramp incremental exercise test was 11.6 ± 1.7 min. The mean V̇O_2max_ of the group was 50.2 ± 5.1 mL·min^–1^·kg^–1^ and the P-V̇O_2max_ corresponded to 334 ± 56 W and 4.7 ± 0.4 W·kg^−1^. The highest P_max_ (1027 ± 212 W; 14.5 ± 2.6 W·kg^−1^) and *v*La_max_ (0.70 ± 0.20 mM·s^–1^) were achieved during the 5-s all-out sprint test for each participant. Peak cadence values obtained from the six all-out efforts performed from 30 to 5 s were 134 ± 8 rpm, 138 ± 8 rpm, 136 ± 7 rpm, 132 ± 9 rpm, 137 ± 8 rpm and 137 ± 8 rpm, respectively. The two-segment linear regression model identified a distinct P-*v*La_BP_ at 795 ± 135 W and 11.2 ± 1.5 W·kg^−1^ (R^2^ = 0.98 ± 0.03; SEE% = 2.90 ± 1.78; *p* ≤ 0.01). This corresponded to a ∆La of 6.50 ± 1.27 mM, a *v*La of 0.50 ± 0.10 mM·s^–1^, and a t-*v*La_BP_ of 13.3 ± 1.9 s (Table 1). Figure 1 shows an individual *v*La_BP_, identified as the intersection point derived from plotting the *v*La values against the P_avg_ from six all-out sprint efforts, obtained from a representative participant.

**Table 1:** Parameters associated with the 5, 10, 15, 20, 25, and 30-s all-out sprints

Variables	Exercise duration						*v*La_BP_	*p*-value	${\upeta}_{{\mathrm{p}}}^{2}$
	5-s sprint	10-s sprint	15-s sprint	20-s sprint	25-s sprint	30-s sprint			
P_max_ (W·kg^−1^)	14.37 ± 2.61	13.78 ± 2.50	13.40 ± 1.99	13.03 ± 1.92^a^	13.03 ± 1.94^a^	12.60 ± 1.47^ab^	13.36 ± 1.92	*p* < 0.001	0.36
P_avg_ (W·kg^−1^)	13.07 ± 2.38	11.93 ± 1.75^a^	10.88 ± 1.44^ab^	10.04 ± 1.37^abc^	9.52 ± 1.15^abcd^	8.64 ± 0.76^abcde^	11.25 ± 1.47^a^	*p* < 0.001	0.84
∆La (mM)	3.49 ± 1.02	5.76 ± 1.44^a^	7.18 ± 1.47^ab^	9.30 ± 1.72^abcd^	11.16 ± 1.50^abcde^	12.22 ± 1.21^abcdef^	6.50 ± 1.27^a^	*p* < 0.001	0.95
*v*La (mM·s^–1^)	0.70 ± 0.20	0.58 ± 0.14^a^	0.48 ± 0.10^ab^	0.47 ± 0.09^ab^	0.45 ± 0.06^ab^	0.41 ± 0.04^ab^	0.50 ± 0.10^a^	*p* < 0.001	0.66

P_max_: the highest instantaneous power production during the sprints; P_avg_: the mean power output measured during the sprints; ∆La: delta blood lactate response; *v*La: the rate of blood lactate accumulation; ${\upeta}_{\mathrm{p}}^{2}$: partial eta squared. Data are presented as mean ± SD.

^a^Significantly different from 5-s sprint, *p* < 0.05

^b^Significantly different from10-s sprint, *p* < 0.05

^c^Significantly different from *v*La_BP_, *p* < 0.05

^d^Significantly different from 15-s sprint, *p* < 0.05

^e^Significantly different from 20-s sprint, *p* < 0.05

^f^Significantly different from 25-s sprint, *p* < 0.05

**Fig. 2:**
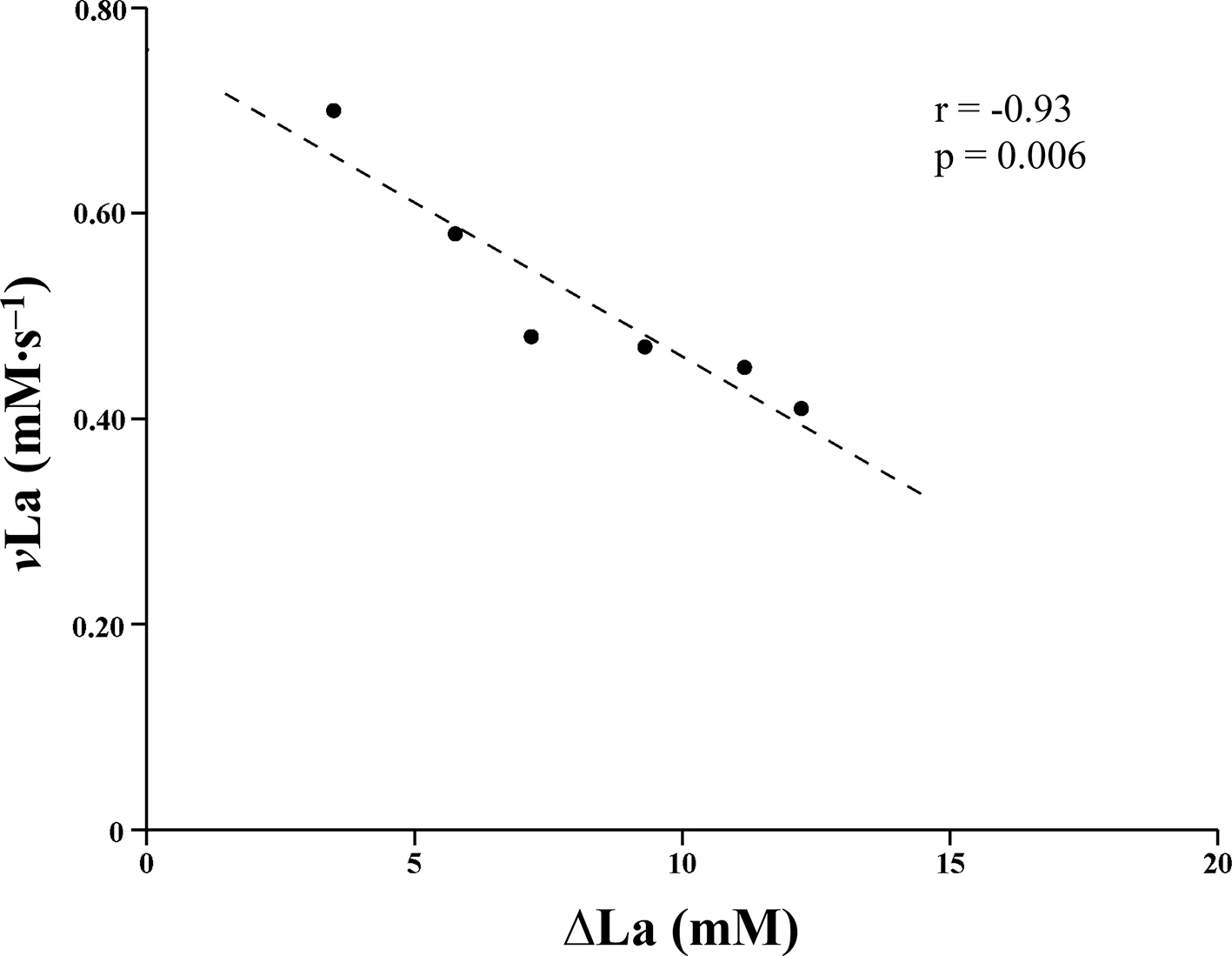
Scatterplot of the relationship between the rate of blood lactate accumulation (*v*La) and change in blood lactate concentration (∆La) based on group-averaged values across six all-out sprints (from 5 to 30 s). The figure shows the very large negative correlation between *v*La and ∆La

There was a significant main effect of sprint duration on ∆La and *v*La (∆La: *p* < 0.001, ${\upeta}_{\mathrm{p}}^{2}$ = 0.95; and *v*La: *p* < 0.001; ${\upeta}_{\mathrm{p}}^{2}$ = 0.66). During the 30 to 15-s all-out sprints, *v*La responses did not increase significantly (0.41 ± 0.04 to 0.48 ± 0.10 mM∙s^−1^; *p* > 0.05); however, a marked increase in *v*La was achieved during the 10-s (0.58 ± 0.14 mM∙s^−1^; *p* < 0.05) and especially the 5-s sprints (0.70 ± 0.20 mM∙s^−1^; p < 0.01). Additionally, there was a significant difference in *v*La between the 10 s and 5 s sprint tests (*p* < 0.05). Figure 2 illustrates the negative correlation between *v*La and ∆La responses across the sprint efforts.

**Fig. 3:**
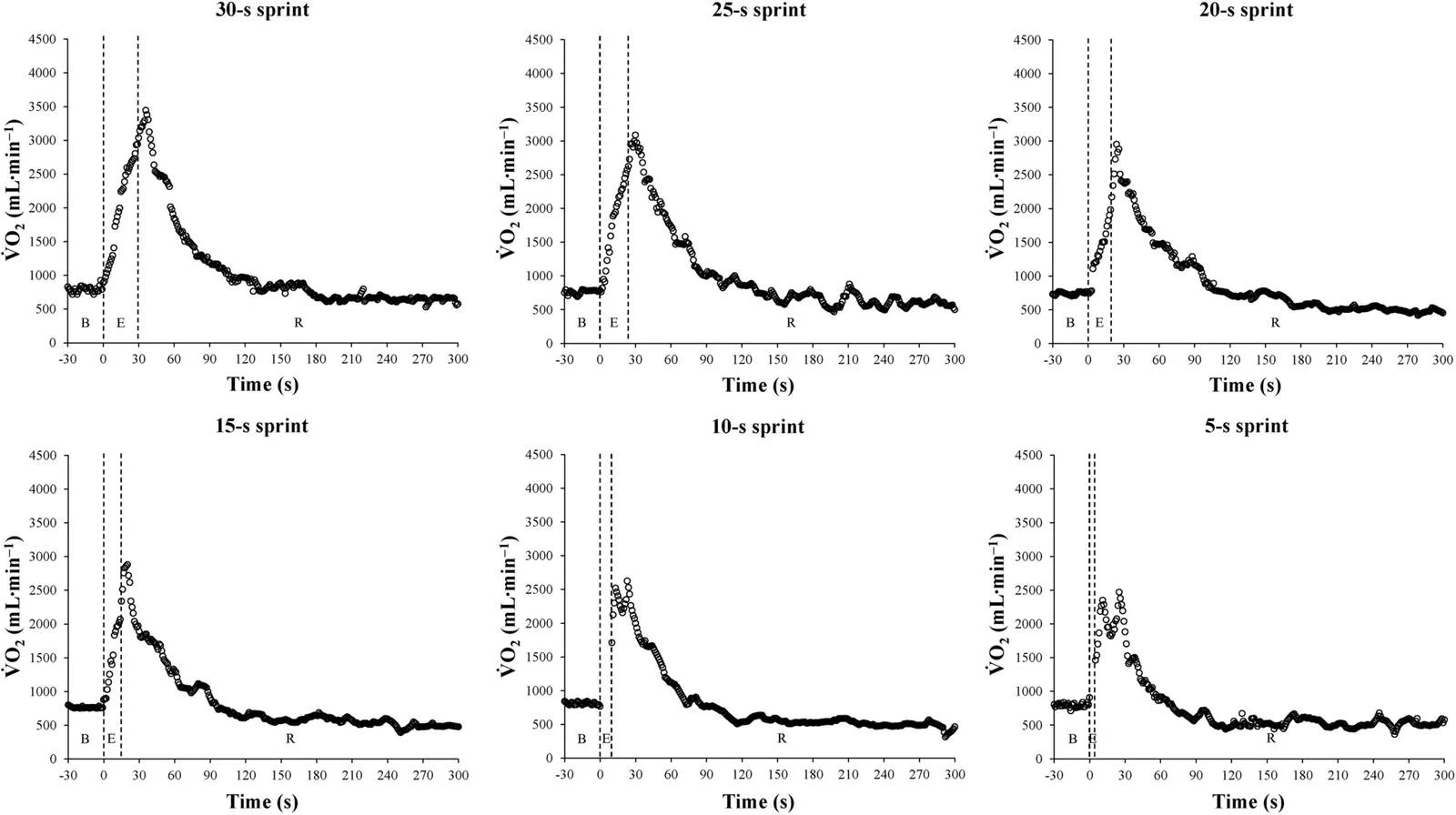
Pulmonary V̇O_2_ responses to six all-out sprint tests performed at durations ranging from 30 to 5 s in a representative participant. Breath-by-breath V̇O_2_ responses were interpolated to 1-s intervals. V̇O_2_ kinetics was characterised by an apparently linear profile during those sprints performed at 15﻿–30 s; however, during 5 and 10-s sprints, participant frequently exhibited breath-holding behaviour, with peak V̇O_2_ typically reached during recovery and displaying an M-shaped profile. B: baseline; E: exercise; R: recovery

V̇O_2_ on-kinetics of the all-out sprints performed below the P-*v*La_BP_ (i.e., for 15-s, 20-s, 25-s and 30-s all-out sprints) was best described by a linear function (R^2^ ≥ 0.96; SEE ≤ 272 mL·min^–1^; *p* < 0.001), while the V̇O_2_ data could not be modelled for all-out sprints performed above the P-*v*La_BP_ (i.e., for 5-s and 10-s all-out sprints). As expected, for the 5-s and 10-s shorter bursts performed above the P-*v*La_BP_, all participants preferred to hold their breath (Fig. 3). Moreover, V̇O_2_ responses continued to increase during the first 5﻿–20 s of recovery periods for the 30, 25, 20, and 15-s sprints. Following the achievement of the peak response, V̇O_2_ decreased exponentially during recovery and were described by double exponential model (R^2^ ≥ 0.96; SEE ≤ 148 mL·min^–1^; *p* < 0.001). Similarly, because the first breath was seen within the first moments of recovery, the highest V̇O_2_ values were always attained within the recovery period following the 5 and 10-s sprints. However, there was an M-shape in V̇O_2_ during the recovery period and those response kinetics could not be modelled by traditional functions following the 5 and 10-s sprints.

## Discussion

The purpose of the present study was to investigate whether individual P-*v*La_BP_ derived from the *v*La–power relationship from six all-out sprint tests corresponds to the boundary which separates the extreme-3 from the extreme-2 subzone. Consistent with our hypothesis, the P-*v*La_BP_ was found to separate a range of power outputs wherein V̇O_2_ kinetics during exercise could be best described with a linear function from a range of power outputs wherein V̇O_2_ kinetics could not be modelled at all. The upper boundary of the extreme-2 intensity exercise zone may therefore be considered to represent the highest all-out effort beyond which the *v*La shifts from limited to rapidly accelerating behaviour. The *v*La_BP_ corresponded to a mean power output of 795 W and 11.2 W·kg^−1^ (i.e., approximately 238% of P-V̇O_2max_ and 78% of P_max_). Accordingly, the extreme-3 exercise zone includes exercise durations up to approximately 13 s.

The extreme-3 intensity exercise zone of our participants had a range of approximately 232 W and 3.3 W·kg^−1^ between P_max_ and P-*v*La_BP_ (i.e., 1027 – 795 W; 14.5 – 11.2 W·kg^−1^). Within this zone, which comprises very short-term maximal efforts, participants typically prefer to hold their breath, and the peak V̇O_2_ is reached during the recovery period, with the recovery V̇O_2_ response resembling an M-shape (Lamarra 1990; Ozkaya et al. 2023). It is known that breath holding during such short-term, extreme intensity exercise reflects the Valsalva manoeuvre, which significantly increases systolic blood pressure and supports the expression of strength and power (Narloch and Brandstater 1995). If the total exercise test duration incorporates a significant period of breath holding, it is not possible to measure or model V̇O_2_ data during exercise (Ozkaya et al. 2023). In contrast, exercise performed at power outputs below the P-*v*La_BP_ transition (i.e., those in the 15﻿–30 s duration range), performed within the extreme-2 exercise zone, resulted in an apparently linear V̇O_2_ response during exercise along with greater metabolic acidosis. It has been previously suggested that the linearity in V̇O_2_ kinetics for extreme-2 exercise may be due to a combination of V̇O_2_ reflecting a very high demand but being constrained by the V̇O_2max_ and the relatively short exercise duration (Ozkaya et al. 2023). Clearly, however, maximal exercise in this time range enables a high rate of anaerobic glycolysis, as reflected by a greater end-exercise blood La concentration.

On the other hand, a recent systematic review fitted four separate bi-exponential models to cycling data, excluding the 3- and 60-s data points, and predicted that the shortest test duration for eliciting *v*La_max_ is approximately 4.5–5 s (Langley et al. 2025). At shorter exercise durations, the time-equivalent accounting for predominantly phosphocreatine (PCr) driven ATP resynthesis with minimal glycolytic activation and negligible blood La accumulation (t_PCr_) is likely to have a disproportionate impact, potentially inflating variability in *v*La_max_ estimates. Indeed, unlike V̇O_2max_, which is typically operationalised as the highest V̇O_2_ value attained within a standardised time interval (i.e., 1-min), *v*La_max_ is calculated by the ∆La divided by sprint duration. As exercise intensity increases and sprint duration shortens, this ratio-based computation may produce a *v*La_max_ value that is disproportionately high and which may not accurately reflect the underlying glycolytic rate. This issue could be considered one of the most important limitations to the ability to obtain a *v*La_max_ response and to use this value as an indicator of maximal glycolytic power. Leaving these debates aside, our findings suggest that, rather than attempting to determine a single *v*La_max_ value, it may be more appropriate to frame the phenomenon as a *v*La breakpoint (i.e., *v*La_BP_), with the corresponding power output representing a transition that separates the extreme-2 and extreme-3 subzones.

A further methodological issue in the *v*La_max_ literature concerns the t_PCr_ and the variability in how it is defined and calculated. It has been claimed that t_PCr_ refers to the beginning of the sprint performance that is dominated by PCr breakdown without a significant activation of glycolysis or accumulation of blood La (Mader 1984, 1994; Heck et al. 2003; Yang et al. 2023). There are different assumptions when calculating the t_PCr_. For example, for the 100 m, 200 m, and 400 m sprints (i.e., approximately 10–12-s, 20–25-s and 45–60-s duration), it is suggested that t_PCr_ be fixed at 3-s, 4-s, and 8-s time periods, respectively (Mader 1994; Heck et al. 2003). Meixner et al. (2024a) suggest the use of a standard t_PCr_ of 3.5 s. In another method, the time to reach P_max_ is considered to be the t_PCr_ duration (Heck et al. 2003; Manunzio et al. 2016). On the other hand, it has also been suggested that the t_PCr_ should be defined as the time window from the onset of exercise to the point at which power output has decreased by 3.5% after the P_max_ is reached (Weber 2003; Heck et al. 2003; Langley et al. 2024). As another example, Yang et al. (2023) have recently suggested that the contribution of oxidative energy metabolism should be taken into account to reveal a more genuine *v*La_max_. However, it is important to note that this time interval approach in which glycolytic activity does not occur is merely hypothetical assumption (Gaitanos et al. 1993; Brooks 2018), and none of these methods has yet gained widespread acceptance or evolved into a broadly endorsed consensus. In a recent review, Wackerhage et al. (2025) highlighted several conceptual and methodological limitations associated with the estimation of *v*La_max_, suggesting that calculating a peak *v*La value (i.e., *v*La_peak_) without subtracting a t_PCr_ may offer a more valid alternative. Indeed, in empirical settings, it has been suggested to assume “t_PCr_ = 0” and to calculate *v*La_max_ by dividing ∆La by test duration (Quittmann 2026), given that phosphagen contribution can only be estimated with limited precision (Hirvonen et al. 1987; Gastin 2001; Quittmann 2026) and that accelerated glycolysis and lactate accumulation begin immediately at exercise onset (Gastin 2001; Brooks et al. 2022; Quittmann 2026). Accordingly, we took this approach in the present study.

From a practical perspective, the P-*v*La_BP_ could be useful in discriminating and prescribing sprint interval training (SIT) and repeated sprint training (RST). Over the past two decades, the physiological and performance benefits of SIT (i.e., 20–30-s all-out sprints, performed at approximately 150–200% of P-V̇O_2max_) have been consistently demonstrated (Burgomaster et al. 2005; Gibala et al. 2006). Similar improvements have been described even following very short-duration, high-intensity intermittent exercise of 8–12 s, i.e., RST (Buchheit and Laursen 2013). In the current study, the t-*v*La_BP_, corresponding to an effort time of approximately 13 s, may be considered a boundary separating SIT and RST prescriptions. Indeed, exercise involving comparatively longer sprint bouts and yielding high end-exercise blood La appears more closely aligned with SIT, while much shorter bouts, often accompanied by breath-holding and characterised by low end-exercise blood La responses despite a high lactate accumulation rate, are consistent with RST.

In terms of limitations, it should be noted that post-exercise La was sampled at the 1st, 3rd, 5th, 7th and 10th min. Future work should sample blood more frequently (e.g., every minute) to refine breakpoint estimation. Additionally, further investigations should consider a broader range of populations, including well-trained or elite athletes, females, and older adults, and should also evaluate the validity of the approach for other exercise modalities. The reliability of *v*La_BP_ determination could also be investigated in future studies.

## Conclusions

The present study identified a P-*v*La_BP_ that separated the power outputs that provide the greatest *v*La from those that generate higher levels of La. This P-*v*La_BP_ boundary was found to occur at approximately 13-s of all-out effort for the physically active participants in this study. The all-out sprints performed below the P-*v*La_BP_ resulted in apparently linear V̇O_2_ response kinetics, while it was not possible to measure or model V̇O_2_ data for the all-out sprints performed above the P-*v*La_BP_. The results of this study may be useful in differentiating extreme-2 from extreme-3 exercise and potentially in the prescription of SIT and RST.

## Data Availability

The datasets generated and/or analysed during the current study are available from the corresponding author on reasonable request.

## Abbreviations

A_fc_: Amplitude of the fast component
A_sc_: Amplitude of the slow component
ES: Effect size
La: Blood lactate concentration
La_peak_: Peak blood La value measured in recovery period
La_rest_: Resting blood La value
P_avg_: Mean power output
P_max_: Highest instantaneous power production
P-*v*La_BP_: Power output corresponding to the *v*La_BP_
P-V̇O_2max_: Power output corresponding to the V̇O_2max_
PCr: Phosphocreatine
RST: Repeated sprint training
R^2^: Coefficient of determination
SD: Standard deviation
SEE: Standard error of estimation
SIT: Sprint interval training
TD_fc_: Time delay of the fast component
TD_sc_: Time delay of the slow component
t_PCr_: Time equivalent to account for energy resynthesis via PCr
t_test_: Sprint duration
t-*v*La_BP_: Exercise duration corresponding to the *v*La_BP_
*v*La: Rate of blood La
*v*La_BP_: Breakpoint in the relationship between the *v*La and power output
*v*La_max_: Maximal *v*La value
*v*La_peak_: Peak *v*La value
V̇O_2_: Pulmonary O_2_ uptake
V̇O_2(base)_: V̇O_2_ obtained from the last 1 min of no-resistance cycling
V̇O_2(t)_: V̇O_2_ responses achievable at any time during exercise
V̇O_2max_: Maximal O_2_ uptake
${\upeta}_{\mathrm{p}}^{2}$: Partial eta squared
τ_fc_: Time constant of the fast component
τ_sc_: Time constant of the slow component
∆La: Change in blood La between the resting and peak
